# Phenotypical Sub-setting of the First Episode of Severe Viral Respiratory Infection Based on Clinical Assessment and Underlying Airway Disease: A Pilot Study

**DOI:** 10.3389/fped.2020.00121

**Published:** 2020-04-02

**Authors:** Maria Arroyo, Kyle Salka, Geovanny F. Perez, Carlos E. Rodríguez-Martínez, Jose A. Castro-Rodriguez, Maria J. Gutierrez, Gustavo Nino

**Affiliations:** ^1^Division of Pediatric Pulmonary and Sleep Medicine. Center for Genetic Research, Children's National Medical Center, George Washington University, Washington, DC, United States; ^2^Department of Pediatrics, School of Medicine, Universidad Nacional de Colombia, Bogota, Colombia; ^3^Department of Pediatric Pulmonology and Pediatric Critical Care Medicine, School of Medicine, Universidad El Bosque, Bogota, Colombia; ^4^Division of Pediatrics, Department of Pediatric Pulmonology, School of Medicine, Pontificia Universidad Catolica de Chile, Santiago, Chile; ^5^Division of Pediatric Allergy and Immunology, Johns Hopkins University, Baltimore, MD, United States

**Keywords:** wheezing, cytokines, respiratory viral, airway immunity, viral bronchiolitis phenotyping

## Abstract

**Introduction:** Viral bronchiolitis is a term often used to group all infants with the first episode of severe viral respiratory infection. However, this term encompasses a collection of different clinical and biological processes. We hypothesized that the first episode of severe viral respiratory infection in infants can be subset into clinical phenotypes with distinct outcomes and underlying airway disease patterns.

**Methods:** We included children (≤2 years old) hospitalized for the first time due to PCR-confirmed viral respiratory infection. All cases were categorized based on primary manifestations (wheezing, sub-costal retractions and hypoxemia) into mild, hypoxemia or wheezing phenotypes. We characterized these phenotypes using lung-X-rays, respiratory outcomes and nasal protein levels of antiviral and type 2 cytokines (IFNγ, IL-10, IL-4, IL-13, IL-1β, and TNFα).

**Results:** A total of 50 young children comprising viral respiratory infection cases (*n* = 41) and uninfected controls (*n* = 9) were included. We found that 22% of viral respiratory infection cases were classified as mild (*n* = 9), 39% as hypoxemia phenotype (*n* = 16) and 39% as wheezing phenotype (*n* = 16). Individuals in the hypoxemia phenotype had more lung opacities, higher probability of PICU admission and prolonged hospitalizations. Subjects in the wheezing phenotype had higher probability of recurrent sick visits. Nasal cytokine profiles showed that individuals with recurrent sick visits in the wheezing phenotype had increased nasal airway levels of type 2 cytokines (IL-13/IL-4).

**Conclusion:** Clinically-based classification of the first episode of severe viral respiratory infection into mild, hypoxemia or wheezing phenotypes provides critical information about respiratory outcomes, lung disease patterns and underlying airway immunobiology.

## Introduction

Viral respiratory infections are the top cause for sick visits and hospitalization during early life (1, 2). However, young children exhibit great variability in the clinical manifestations of viral respiratory infections. In general, these illnesses are self-limited and managed conservatively at home. Nonetheless, some infants have acute respiratory failure and others develop recurrent wheezing illnesses after an initial viral infection (1–3). Despite the enormous clinical variability of this condition, in pediatrics the first episode of viral respiratory infection causing significant respiratory compromise is usually defined as “viral bronchiolitis” (1). Notably, the clinical diagnostic criteria of viral bronchiolitis vary across different geographical locations. While in North America the presence of wheeze in infants aged up to 24 months is the main criterion used to define bronchiolitis (4), in Europe, the presence of inspiratory crackles in infants aged up to 12 months is a common diagnostic criterion (5). These differences in the definition of viral bronchiolitis cause many problems in the management of young children with viral respiratory infections (6). As a result, there is increasing controversy with this term because it combines a constellation of clinical manifestations in an umbrella respiratory syndrome (7–9). The failure of numerous clinical trials using this “one-size-fits-all” definition suggests that viral bronchiolitis is not a single entity but rather a group of disease subsets representing distinct clinical and biological processes (7–9).

Prior studies examining the heterogeneity of viral bronchiolitis have used multidimensional approaches to integrate molecular factors such as viral pathogens, immune responses and microbiome followed by unbiased clustering and/or machine learning algorithms (10, 11). These approaches have provided valuable insights into potential individual mechanisms of disease in viral respiratory infections in young children. However, no prior study examining the heterogeneity of viral bronchiolitis has used the initial bedside respiratory assessment as the key driving variable to classify the first episode of severe viral respiratory infection in infants. Notwithstanding the importance of investigating the molecular biology of this condition, classifying viral respiratory infections according to the individual phenotypical manifestations is probably the most clinically relevant approach to subset this condition.

The overarching goal of this pilot study was to propose that a rigorous clinical assessment enables the stratification and potential prediction of outcomes in infants with the first episode of severe viral respiratory infection. Specifically, we tested the hypothesis that in young children (≤2 years old) hospitalized with the first episode of viral respiratory infection, the bedside assessment of basic clinical respiratory findings (wheezing, subcostal retractions, or hypoxemia) allows an initial phenotypical sub-setting with distinct outcomes and underlying airway disease based on lung-X-rays and nasal cytokine profiles.

## Methods

### Study Design

In this single-center observational study we included full term young children (≤2 years of age) hospitalized for the first time due to PCR-confirmed viral respiratory infection at Children's National Health System (CNHS) in Washington DC. We used age-matched individuals without viral respiratory infection (negative viral PCR) as a control group (for nasal cytokines studies only). Subjects were enrolled from 2014 to 2017. For our viral respiratory infection cases we included only those children hospitalized that had: (1) positive PCR for any of the viruses included in our panel, including rhinovirus (RV), respiratory syncytial virus (RSV), human metapneumovirus (HMPV), influenza A/B, parainfluenza 1–3, and adenovirus; (2) had continued clinical care in CNHS for at least 12 months after discharge; and (3) had available electronic medical record (EMR) data (inpatient and outpatient) to ascertain predictors of interest and main outcomes. We excluded cases with incomplete EMR data and children with: (1) prior respiratory hospitalizations or respiratory sick visits, (2) prior or current use of systemic or inhaled steroids, (3) congenital conditions (e.g., pulmonary abnormalities, cyanotic heart disease, cystic fibrosis, airway abnormalities), (4) immunodeficiency, (5) prematurity (<37 weeks gestational age) and (6) neuromuscular disorders. All clinical and demographic variables including age, sex, and self-reported race/ethnicity were obtained by reviewing EMR at CNHS. We only analyzed nasal specimens obtained for clinical purposes in CNHS. The Institutional Review Board (IRB) of Children's National Medical Center, Washington D.C. approved the study and granted a waiver of informed consent given that this research involved materials collected solely for non-research purposes (clinical indications).

### Nasal Cytokine Measurements

Nasal airway secretions were collected for clinical purposes (viral respiratory detection) using the same standard protocol. Secretions were aliquoted in 1–3 separate tubes and stored at −80°C until further analysis. Nasal airway protein levels of IFNγ, IL-10, IL-13, IL-1β, IL-4, and TNFα were quantified with electrochemiluminescence (MesoScale Discovery, MSD, Rockville, MD). Individuals with values below the lower limit of detection (LLD) were not excluded to prevent selection bias since LLD values were more likely in the groups with lower cytokine responses (e.g., controls). Samples below LLD were assigned the LLD value provided by the manufacturers' instructions: IFNγ= 0.2 pg/ml, IL-10 = 0.03 pg/ml, IL-13 =0.24 pg/ml, IL-1β = 0.15, IL-4 = 0.02 pg/ml, and TNFα = 0.51 pg/ml.

### Clinical Variables

Clinical data collection was through EMR review conducted by research staff blinded to group assignment (phenotypes), clinical outcomes and nasal cytokine data. We recorded clinical features at presentation such as sub-costal retractions, wheezing and hypoxemia (defined as need of supplemental O2), respiratory rate (RR), heart rate (HR), length of stay (LOS), need for pediatric critical care unit (PICU) admission and the binary presence (0 or ≥1 episode) of a respiratory illnesses leading to hospitalization or emergency department (ED) visit within 12 months after the index hospitalization. We only counted as respiratory hospitalization or ED visits those in which the primary complaint was any type of respiratory sign or symptom (e.g., cough, nasal/chest congestion, wheezing, respiratory distress, hypoxemia, etc.).

### Respiratory Assessment and Phenotypes Definition

We used the binary presence or absence during the entire hospitalization of hypoxemia, wheezing and/or sub-costal retractions (clinical marker of hyperinflation) (12–15), to define: (1) a “mild phenotype” composed by children with viral respiratory infection hospitalized due to persistent symptoms (e.g., cough, poor oral intake) but without hypoxemia, wheezing or sub-costal retractions, (2) a “hypoxemia phenotype” characterized by the need for supplemental O2, and (3) a “wheezing phenotype” characterized by wheezing or sub-costal retractions in the absence of hypoxemia. Individuals with both markers of lower airway obstruction (wheezing and sub-costal retractions) were considered part of the wheezing phenotype regardless of hypoxemia.

### Lung Imaging Analysis

The initial chest radiograph (CXR) performed during hospitalization was used for visual scoring of focal opacities (alveolar infiltrates), conducted blindly and independently by two pediatric pulmonologist (GN and MA). Prior to scoring we automatically generated four zones with a weighted-shape partitioning pediatric lung segmentation and image intensity standardization algorithm, as described (16–18). Then we visually scored (binary 1,0) the presence of focal opacities per zone and quantified the number of zones affected in the lungs (0–4; upper/lower each lung).

### Statistical Analysis

Differences between groups on continuous variables were analyzed using the unpaired *t*-test, the Mann-Whitney *U*-test, or one-way analysis of variance for continuous variables whichever was appropriate. Associations between categorical variables were analyzed using the X2 test, the Fisher's exact test or logistic regression, whichever was appropriate. Given the range of values for the cytokines, they were log10-transformed for statistical analysis. The data were analyzed with the Minitab Statistical Package V.18.1. (Minitab, Inc., State College, PA).

## Results

### Study Subjects and Clinical Phenotypes of Severe Viral Respiratory Infections

We included a total of 50 young children (≤2 years old) in this study. Based on their clinical presentation all subjects hospitalized with PCR-confirmed viral respiratory infection (*n* = 41) were sub-classified into three groups: mild phenotype, hypoxemia phenotype and wheezing phenotype (see methods). Table 1 shows baseline characteristics of all study subjects and clinical features of each phenotype. We found that 22% of viral respiratory infection cases included were classified as mild (*n* = 9), 39% as hypoxemia phenotype (*n* = 16) and 39% as wheezing phenotype (*n* = 16). The median age at recruitment of all the study subjects included was 10.6 months (IQR 12.2), 31 (62%) were male and 24 (48%) were black race/ethnicity. We did not identify significant differences in age, gender and race/ethnicity among the study groups (Table 1). Rhinovirus (RV) was the most common viral pathogen in the mild phenotype (Table 1). Based on pre-set definitions (see methods) none of the individuals in the mild phenotype had wheezing, sub-costal retractions or hypoxemia and none of the subjects in the hypoxemia phenotype had both wheezing and sub-costal retractions. In the wheezing phenotype, half of the subjects required supplemental O2, 69% (11/16) had both wheezing and sub-costal retractions, 75% (12/16) had wheezing alone, and 94% (15/16) had sub-costal retractions alone. We did not find significant differences in respiratory rate or heart rate among the clinical phenotypes, however, they trended to be lower in the mild group (Table 1).

**Table 1:** Baseline characteristics of all study subjects and clinical features of each viral respiratory infection phenotype.

**Individual characteristics**	**Control uninfected (*n* = 9)**	**Mild phenotype (*n* = 9)**	**Hypoxemia phenotype (*n* = 16)**	**Wheezing phenotype (*n* = 16)**	***P*-value^a^**
Age in months, median (IQR)	11.4 (17.16)	12.12 (13.26)	12.78 (12.24)	6.36 (8.64)	0.16
Male, *n* (%)	5 (56)	7 (78)	10 (63)	9 (56)	0.70
Black, *n* (%)	6 (67)	3 (30)	7 (44)	8 (50)	0.28
Family history of asthma, *n* (%)	2 (22)	3 (30)	2 (13)	6 (38)	0.36
**Viral pathogen**					
RV, *n* (%)	-	8 (89)	8 (50)	6 (38)	0.03
RSV, *n* (%)	-	1 (11)	5 (31)	8 (50)	0.11
Mixed, *n* (%)	-	4 (44)	2 (13)	5 (31)	0.18
HMPV, *n* (%)	-	2 (22)	1 (6)	4 (25)	0.29
Adenovirus, *n* (%)	-	3 (30)	1(6)	1(6)	0.14
Parainfluenza, *n* (%)	-	0 (0)	0 (0)	1(6)	-
Influenza, *n* (%)	-	0 (0)	3 (19)	1(6)	-
**Clinical presentation**					
Need for supplemental O2, *n* (%)	-	0 (0)	16 (100)	8 (50)	-
Wheezing and sub-costal retractions, *n* (%)		0 (0)	0 (0)	11 (69)	-
Wheezing alone, *n* (%)	-	0 (0)	1 (6)	12 (75)	-
Sub-costal retractions alone, *n* (%)	-	0 (0)	9 (56)	15 (94)	-
Respiratory rate, mean (*SD*)	-	39 (9)	43 (12)	45 (9)	0.32
Heart rate, mean (*SD*)	-	143 (29)	153 (31)	162 (24)	0.28

* *p-values obtained by one-way analysis of variance for continuous variables and by logistic regression for binary variables*.

### Clinical Phenotypes of Severe Viral Respiratory Infection and Findings in X-Ray Lung Imaging

**Figure 1:**
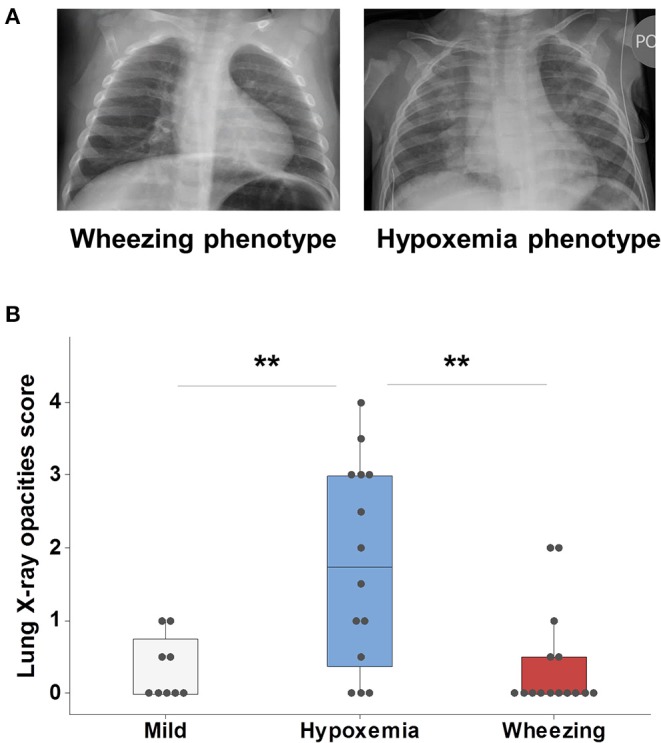
Clinical phenotypes of severe viral respiratory infection using X-ray lung imaging. **(A)** Comparison of two cases of RSV infection; the case in the left side (wheezing phenotype) had wheezing/subcostal retractions and a CXR with increased perihilar markings and hyperinflation; the case in the right side (hypoxemia phenotype) had supplemental O2 needs and a CXR with multifocal alveolar opacities (right>left). **(B)** Analysis of all viral respiratory infection cases with available CXRs (*n* = 38) demonstrated that individuals with a hypoxemia phenotype had more lung-X ray opacities. Boxes represent the 25 and 75th percentiles, **p < 0.01.

We used blinded scoring of CXRs as a separate marker of lung disease during the first viral respiratory infection hospitalization to further characterize our clinical phenotypes (Figure 1A). All children admitted due to viral respiratory infection had CXR available for review except for two cases in the hypoxemia phenotype and one in the wheezing phenotype. In this study we focused only on the number of quadrants affected by opacities (0–4) and we observed significant agreement using this approach (83% Cohen's Kappa). As shown in Figure 1, we found that individuals with a hypoxemia phenotype had significantly more lung zones affected (median = 1.75, IQR = 2.6) than those classified as mild (median = 0, IQR = 0.75) or wheezing phenotypes (median = 0, IQR = 0.5, Figure 1B). These data supported the proposed respiratory phenotypes as a valid method to classify the first episode of severe viral respiratory infection based on clinical manifestations and CXR disease patterns.

### Clinical Phenotypes of Severe Viral Respiratory Infection and Respiratory Outcomes

**Figure 2:**
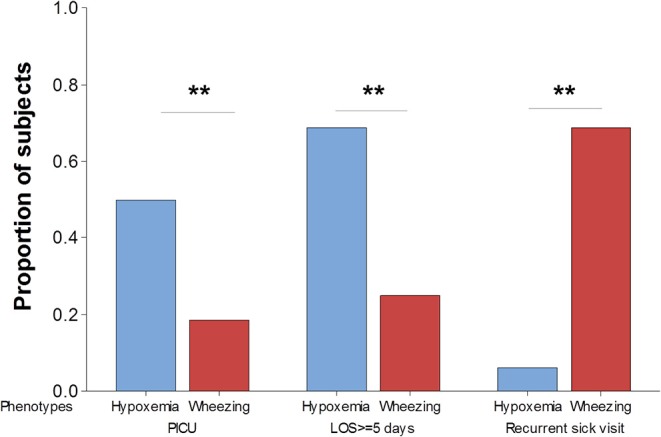
Clinical phenotypes of severe viral respiratory infection using respiratory outcomes. The probability of requiring transferred to pediatric intensive care unit (PICU), prolonged hospitalization defined as ≥5 days length of staying (LOS) and recurrent respiratory sick visits after discharge are significantly different in children hypoxemia (blue) vs. wheezing (red) phenotypes. **p < 0.01.

We next examined if the clinical phenotypes of the first viral respiratory infection requiring hospitalization are linked to different clinical outcomes. For these analyses we used three clinically relevant outcomes: (1) transfer to pediatric intensive care unit (PICU) for advanced respiratory support; (2) length of hospitalization; and (3) recurrence defined as ≥1 subsequent respiratory sick visit (ED or hospitalization) within 12-months of discharge. As expected, none of the subjects in the mild phenotype required transferred to PICU and they had relatively short admissions (median 3 days, IQR 6.5). In contrast, individuals in the hypoxemia phenotype had significantly higher probability of having PICU admission or a prolonged hospitalization (≥ 5 days) than those in the wheezing phenotype (Figure 2). Interestingly, as shown in Figure 2, individuals in the wheezing phenotype had a significantly higher probability of respiratory sick visits after discharge (69%, 11/16) relative to subjects in the hypoxemia phenotype (6%, 1/16), or those in the mild phenotype (22%, 2/9). These data demonstrated that the proposed respiratory phenotypes may be useful to predict clinically relevant outcomes of the first episode of severe viral respiratory infection hospitalization.

### Clinical Phenotypes of Severe Viral Respiratory Infection and Airway Immune Responses

**Figure 3:**
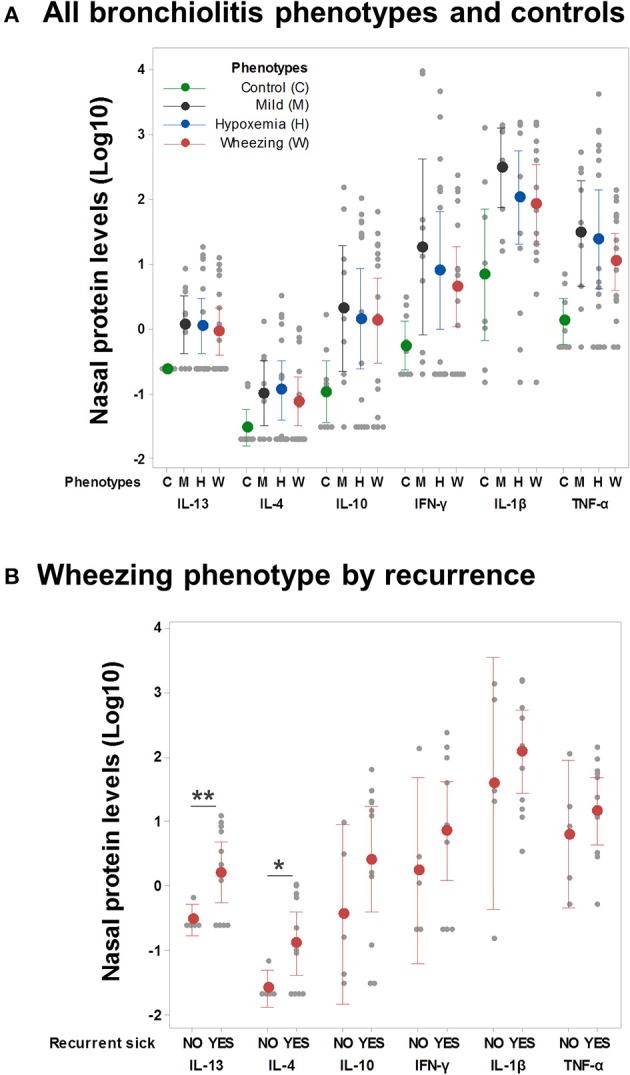
Airway immune responses and phenotype-specific outcomes. **(A)** Nasal cytokine profiles (protein levels pg/ml, log-10 transformed) among controls and individuals in all clinical phenotypes (*n* = 50). Ninety-five percent confidence intervals correspond to ANOVA and Dunn's *post-test* adjusted values. **(B)** Nasal cytokine profiles in the wheezing phenotype only (*n* = 16) according to the presence of recurrent respiratory sick visits after discharge. **p < 0.01, *p < 0.05.

We also compared nasal cytokine profiles (protein levels) among the clinical phenotypes and according to respiratory outcomes. As shown in Figure 3, cytokine levels were overall higher in individuals with viral respiratory infections relative to uninfected controls but we did not find significant differences among clinical phenotypes (Figure 3A). However, children with a wheezing phenotype and ≥1 recurrent respiratory sick visit after discharge had higher nasal airway levels of type 2 cytokines (IL-13/IL-4) without significant differences in other cytokines (Figure 3B). We did not identify other differences in nasal cytokine profiles according to phenotype-specific outcomes in the mild, wheezing or hypoxemia phenotypes (data not shown).

## Discussion

The results of this small pilot study indicated that infants hospitalized with severe viral respiratory infection can be potentially subdivided into clinically-oriented phenotypes. Specifically, we categorized the first hospitalization due to viral respiratory infection based on primary clinical manifestations (wheezing, sub-costal retractions and hypoxemia) as mild, hypoxemia and wheezing phenotypes and then characterized these respiratory phenotypes using lung X-ray imaging, outcomes and nasal antiviral and type 2 cytokines. The main findings are that: (1) Individuals in the hypoxemia phenotype had more lung opacities and higher probability of PICU admission as well as prolonged hospitalization (≥5 days); (2) subjects in the wheezing phenotype had higher probability of recurrent respiratory sick visits; and (3) nasal cytokine profiles showed that individuals with recurrent respiratory sick visits in the wheezing phenotype had enhanced nasal airway levels of type 2 cytokines (IL-13/IL-4). Collectively, these pilot data indicate that a clinically-oriented sub-setting of the first episode of severe viral respiratory infection into distinct phenotypes may provide critical information about respiratory outcomes prediction and airway disease patterns.

Viral bronchiolitis is a term that is often used to group all infants with the first episode of a viral respiratory infection. However, our study supports the prevailing notion that viral bronchiolitis is not a single entity (7–9). Prior studies have identified multiple clinical phenotypes of preschool wheezing (19), but the clinical-driven phenotypes of the first episode of severe viral respiratory infection are still unclear. Based on our current results, the outcome of interest in infants having the first presentation of viral respiratory infection with a wheezing phenotype should be recurrent respiratory symptoms after discharge. We found that young children with wheezing phenotype did not have prolonged hospitalizations or high probability of requiring PICU, however, they did have high risk of recurrent respiratory sick visits, particularly those with high airway type 2 responses (high nasal airway IL-13/IL-4 secretion). The later resembles the pathobiology of asthma (20), and has been identified in some viral bronchiolitis sub-sets with recurrent viral-induced wheezing (21, 22). Thus, asthma therapies (β2 agonist bronchodilators and steroids) might be indicated in this group of young children hospitalized for the first time with a viral respiratory infection (23–25). In contrast, infants with hypoxemia as primary manifestation of the first episode of severe viral respiratory infection are more likely to have ventilation/perfusion mismatch and/or diffusion abnormalities due to lung parenchyma compromise, which is less likely to reoccur in otherwise healthy subjects. As a result, interventions in infants with viral respiratory infections demonstrating a hypoxemia phenotype should aim at reducing acute morbidity and mortality because they had prolonged admissions and high risk of requiring PICU hospitalization.

We also identified a mild respiratory phenotype that likely represents an infection limited to the upper respiratory tract and/or a mild compromise of the lower respiratory tract without causing distress (e.g., hypoxemia or retractions). Although this mild phenotype is probably common in the outpatient setting, it was relatively infrequent in our study (22%) because we only included hospitalized children. Notably, this mild phenotype has been described before in two large cohorts of children hospitalized with bronchiolitis in the USA and in Finland (10). Investigators found that this mild phenotype (denominated “Profile D”) accounted for 17% of hospitalized cases and it was characterized by non-wheezing children without retractions, and a shorter hospital length-of-stay (10).

A potential concern regarding our definition of clinical respiratory phenotypes is that it might be perceived as based on subjective criteria that could overlap. In this regard, it is important to emphasize that the presence or absence of basic clinical findings (wheezing, subcostal retractions or hypoxemia) and the detection of opacities in CXR should not be considered subjective criteria. These robust clinical assessments are used routinely in clinical practice. In addition, in this study we decided to use binary classifications to avoid subjective severity grading (e.g., mild vs. moderate) and we did not include more detailed descriptions of clinical findings (e.g., auscultatory rales) or lung imaging (e.g., morphological assessments of opacities or bronchial markings) to minimize subjective assessments of viral respiratory infections. To resolve the overlap of clinical findings we used a systematic approach to categorize each case of viral respiratory infection depending on the presence or absence of wheezing, subcostal retractions or hypoxemia (described in methods). This systematic approach was initially hypothesized based on the pathobiology of viral respiratory infections given that in some individuals airway hyperreactivity (e.g., wheezing) may predominate over alveolar gas exchange issues. It is important to highlight that this initial hypothesis was tested by conducting studies to specifically prove the validity of the systematic approach proposed. Indeed, as shown in Figure 2, we found that each of the respiratory phenotypes had statistically significant different lung X-ray patterns and clinical endpoints. Of note, clinical and lung imaging data was generated by research staff blinded to group assignment (phenotypes), clinical outcomes and nasal cytokine data. Thus, our pilot data suggest that a systematic clinical assessment may enable the stratification and potential prediction of outcomes in infants with the first episode of severe viral respiratory infection.

Our study has some limitations. First, it is very important to emphasize that this is a small pilot study and our findings need prospective validation. We had enough power to test our main hypotheses (differences among respiratory phenotypes), but the small sample size did not allow us to consider the role of breastfeeding, viral pathogen, eczema, atopy/allergy, socioeconomic status and environmental factors (e.g., smoking and daycare attendance). It is also possible that the small sample size may have prevented us from identifying additional differences in clinical parameters (e.g., respiratory rate) or airway cytokine profiles among our study groups. We also recommend limiting the generalization of this pilot data. Indeed, although the mean IL-13/IL-4 levels were elevated in the recurrent wheeze phenotype, this was not the case for all individuals in this group indicating that there is substantial heterogeneity in the pathobiology of wheezing illnesses during viral respiratory infections in young children.

A second limitation was that all clinical data was collected via EMR chart review instead of in-person encounters and without parental questionnaires that could have ensured a more reliable documentation of clinical outcomes after discharge. Our EMR system provides automated options to document complete clinical parameters and physical findings, however, we believe our clinical respiratory phenotypes require validation using a prospective design with additional physical examination findings that are considered in some viral bronchiolitis definitions (e.g., rales), and variations on respiratory manifestations depending on the stage of illness.

In summary, although further validation is required, the impact of our brief research report is that it presents the initial description of new viral respiratory clinical phenotypes in young children with different outcomes and airway disease patterns. Specifically, our pilot results indicated that: (1) the clinically-based phenotyping of the first episode of severe viral respiratory infection may provide critical information about respiratory outcomes (e.g., PICU admission or recurrence after discharge) and airway disease patterns (e.g., lung infiltrates) that may not be obtained by analysis of airway molecular biomarkers alone (e.g., nasal cytokine profiles); and (2) the correlation of airway molecular profiles (e.g., type 2 cytokines) with clinical outcomes in viral respiratory infections may need to take into consideration individual clinical phenotypes (e.g., wheezing phenotype). Given that viral respiratory infections are still a major health problem worldwide, we feel larger longitudinal studies are urgently needed to further define specific clinical respiratory phenotypes and to discover new mechanisms of disease and phenotype-specific interventions.

## Data Availability Statement

The datasets generated for this study are available on request to the corresponding author.

## Ethics Statement

The studies involving human participants were reviewed and approved by IRB Children's National Medical Center, Washington DC. Written informed consent for participation was not provided by the participants' legal guardians/next of kin as the Institutional Review Board (IRB) granted a waiver of informed consent given that this research involved materials collected solely for non-research purposes (electronic medical records and nasal samples obtained for clinical indications).

## Author Contributions

GN, KS, MA, GP, CR-M, JC-R, and MG contributed to the design and implementation of the research, to the analysis of the results and to the writing of the manuscript.

### Conflict of Interest

The authors declare that the research was conducted in the absence of any commercial or financial relationships that could be construed as a potential conflict of interest.
